# Comparative greenness assessment for the simultaneous estimation of diclofenac and methocarbamol in their tablets applying synchronous fluorimetry

**DOI:** 10.1038/s41598-026-41615-y

**Published:** 2026-03-22

**Authors:** Maram Attia, Ghada M. Hadad, Randa A. Abdel Salam, Mary E. K. Wahba

**Affiliations:** 1 Pharmaceutical Analytical Chemistry Department, Faculty of Pharmacy, Suez Canal University, Ismailia, Egypt; 2 Pharmaceutical Chemistry Department, Faculty of Pharmacy, Delta University for Science and Technology, Gamasa, Egypt

**Keywords:** Diclofenac, methocarbamol, synchronous fluorescence spectroscopy, validation, Chemistry, Drug discovery, Medical research

## Abstract

For treatment of muscle spasms associated pain, combination of nonsteroidal anti-inflammatory drugs like diclofenac (DIC) and muscle relaxants as methocarbamol (MET) is usually utilized. This work represents a novel, rapid, facile, sensitive, and selective first derivative synchronous fluorescence spectroscopy (FDSFS) for the simultaneous determination of DIC and MET in their combined tablets. Factors influencing method’s sensitivity were investigated, and the best findings were accomplished applying Δ λ = 60 nm and using water as a diluting solvent. Through applying the optimized experimental conditions, DIC showed a lower detection limit of 0.15 µg/mL and a quantitation limit of 0.30 µg/mL, while MET corresponding values were 0.03 µg/mL and 0.05 µg/mL. Diclofenac was measured at 288 nm, while methocarbamol was measured at 346 nm, exhibiting linearity over the concentration ranges of 0.3–2.5 and 0.05–5.0 µg/mL, respectively. Through application to several laboratory-prepared mixtures and commercial formulation, the suggested method’s applicability was determined. When comparing the proposed method to the reported HPLC method using the student’s *t*-test and *F*-ratio test, no discernible differences were found. Due to simplicity and economical advantage of the method, it can be applied in quality control laboratories for analysis of the studied drugs. The evaluation of the method’s eco-friendliness and greenness was also performed using Analytical GREEnness (AGREE), Green Analytical Procedure Index (GAPI) and Analytical Green Star Area (AGSA) metrics. Complete validation procedures were applied to the suggested approach in compliance with the International Conference on Harmonization’s criteria.

## Introduction

**Fig. 1:**
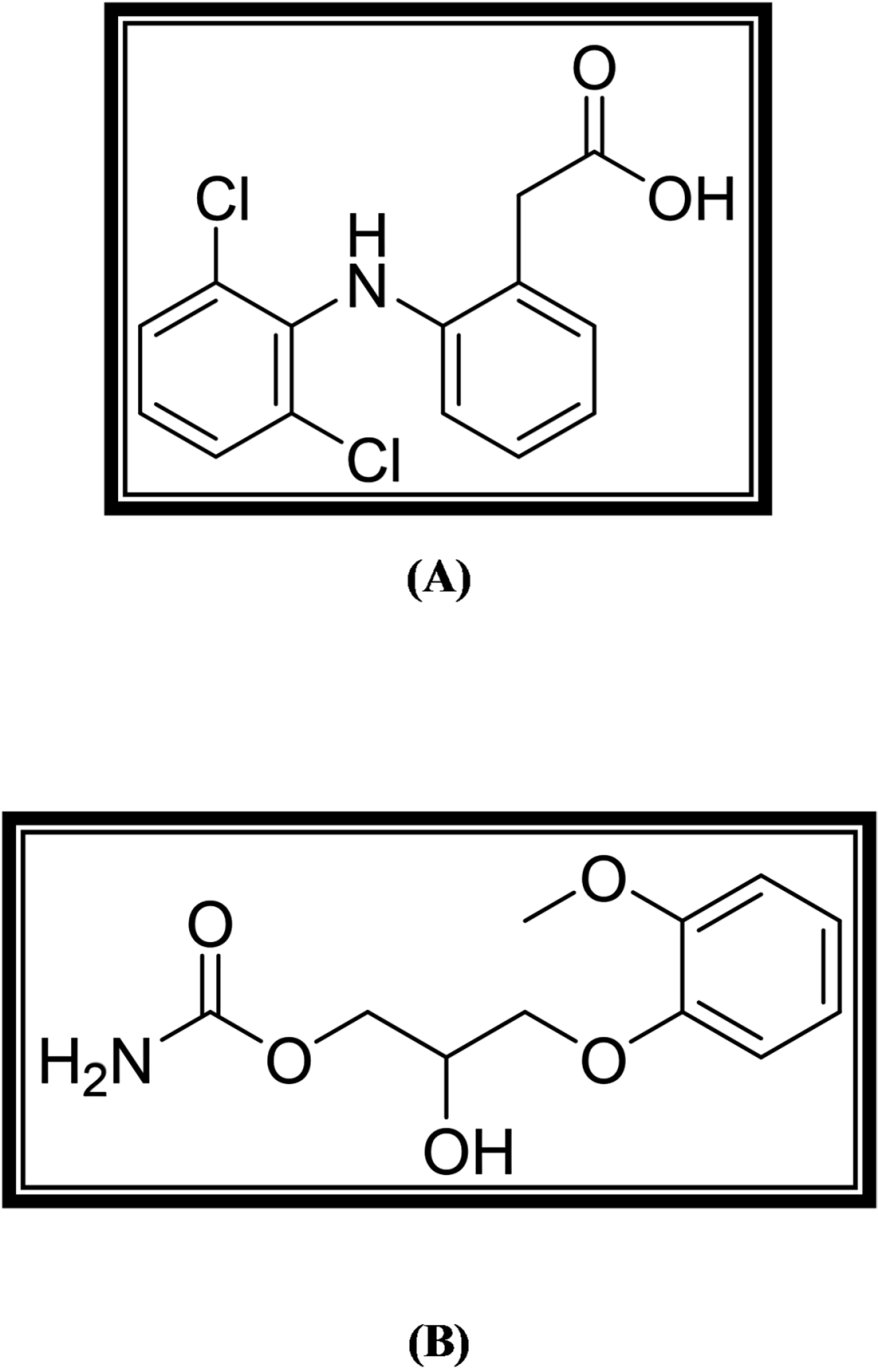
Structural formulae of DIC (**A**) and MET (**B**).

Diclofenac (DIC) is a phenyl acetic acid derivative, chemically designated as [2-(2,6-Dichloroanilino) phenyl] acetic acid^1^ (Fig. 1A). As a non-steroidal anti-inflammatory medication^2^, it acts by blocking the cyclooxygenase 1 and 2 enzymes, which prevents prostaglandin synthesis^3^. It is regarded as a first-line treatment for rheumatoid arthritis, osteoarthritis, and other illnesses that cause both acute and chronic pain and inflammation^4,5^.

Methocarbamol (MET); a guaiacol glyceryl ether (Fig. 1B) [2-hydroxy-3-(2-methoxyphenoxy) propyl] carbamate^1^. A centrally-acting skeletal muscle relaxant with an unclear mode of action^6–8^. Methocarbamol is used to treat muscle spasms in individuals with pyramidal spine abnormalities, such as ruptured intervertebral discs^9^.

Several techniques, such as spectrophotometry^10–16^, gas chromatography^17–21^, high performance liquid chromatography^22–29^, thin layer chromatography^30–33^ and ultra performance liquid chromatography^34,35^ were reported in earlier studies to quantify DIC and MET wheather alone or in conjunction with other medications.

The routine quality control analysis of the drugs in concern still requires an alternative to these techniques, as liquid chromatography needs skilled analysts to operate, constant maintenance, uses costly, high-grade chemicals, and consumes a lot of organic solvents which deprive it from the required sustainability, and spectrophotometry’s sensitivity is low. These facts gave us the motivation to carry out this work and because up to the moment, no analytical tool has been reported for the simultaneous determination of the concerned drugs using a direct fluorescence spectroscopic method which is advantageous by being rapid, sensitive, economic, and nondestructive requiring minimal sample preparation. However, the analysis of drug mixtures by single-wavelength spectrofluorimetry is limited since their spectra overlap^36^. This limitation is overcomed by synchronous fluorescence spectroscopy (SFS), which reduces overlap between the resulting spectra, improves selectivity, and narrows spectral bands^37^. Variable angle synchronous luminescence, constant energy, and constant wavelength are some of the several modalities of SFS methods. Constant-energy SFS (CESFS), proposed by Inman et al.^38^. In CESFS, the excitation and the emission monochromators are scanned synchronized so that a constant energy difference is maintained between two monochromators. This technique significantly increased the selectivity of the analysis of PAHs mixtures^39^. Another technique is to quantify fluorescence by simultaneously scanning the excitation and the emission monochromators at various rates. This technique, known as variable-angle SFS (VASFS), is known for both its great selectivity and versatility^40,41^. Because of its special characteristics, constant wavelength synchronous fluorescence spectroscopy (CWSFS) was used in this study. It entails scanning both the excitation and emission wavelengths simultaneously at constant Δλ values^37^. Synchronous fluorimetry in conjunction with derivatization is beneficial in terms of sensitivity and selectivity; it frequently yields peaks that are well-resolved in multicomponent mixes, simplifies intricate spectra, and produces narrow-width, sharp bands^42^, as well as high tolerance limits and recovery percentages. For these reasons, it was adopted in this work^42^.

## Experimental

### Apparatus

Shimadzu RF-6000 (A40245801833SA) Spectro fluorophotometer equipped with a 150 W Xenon arc lamp, (Shimadzu, Kyoto, Japan). The pH was adjusted using a “Jenway 3503 digital pH meter (Stone, Staffs, UK)”. “Stakpure Pure water system OmniaTap12 UV” used to get deionized water. “Sartorius Entris 224-1S laboratory balance” used to weigh the raw materials of drugs.

### Materials and reagents

Diclofenac acid of purity (100.85%), (Batch #678) was generously supplied by Swiss pharma Co., Ciba-Geigy, Switzerland and Methocarbamol of purity (99.98%), (Batch# B/MC-IPA/12/20/010) was kindly gifted by (MUP); Medical Union Pharmaceuticals Co., Cairo, Egypt. Purity of both drugs was determined by applying the methods published by United States Pharmacopeia (USP). Methoquick^®^ tablets: 50 mg DIC and 500 mg MET/ tablet, (Batch #240381), manufactured by (Sigma pharmaceutical & chemical industries, Egypt), were obtained from local pharmacies.

Methanol, acetone and acetonitrile (99.9% purity, a product of Fisher scientific, UK) HPLC grade. Potassium dihydrogen phosphate obtained from PIOCHEM Laboratory Chemicals, 1st industrial zone, 6th of October, Egypt.

### Standard stock solutions

Serial dilution was performed with methanol to reach stock solutions of concentrations 100.0 µg/mL. Methanol was used to further dilute the DIC and MET working standard solutions. This solution remained stable in the refrigerator for at least a week.

### Construction of calibration curves

Appropriate volumes of 0.3, 0.5,1.0, 1.5, 2.0 and 2.5 mL for DIC and 0.05, 0.25, 0.5, 1.0, 2.0, 3.0, 3.5, 4.0, 5.0 mL for MET of stock solutions of concentration 100 µg/mL were transferred to 10.0 mL volumetric flasks, 1 mL phosphate buffer (pH 7) was added to each flask then the volume was completed to the mark with deionized water as a diluent. These concentrations covering the linearity ranges of 0.3–2.5 and 0.05–5.0 µg/mL for DIC and MET, respectively, were prepared to construct the calibration curve. Monochromators were scanned at (Δλ) 60 nm to record synchronous fluorescence spectra. The excitation and emission monochromators’ bandwidth were adjusted at 5 nm using a scan rate of 6000 nm/min. A blank experiment was conducted concurrently with each measurement. Shimadzu Lab Solution RF^®^ software was then used to derivatize the resulting SFS spectra. DIC was measured selectively at 288 nm (zero crossing point for MET), while MET could be estimated at 346 nm (zero crossing point for DIC) applying FDSFS. By plotting the corrected peak amplitudes *versus* the final drug concentration (µg/mL), the calibration graphs were established, or the appropriate regression equations were determined.

### Analysis of laboratory prepared mixtures

The marketed formulation’s ratio as well as various laboratory- prepared mixtures with varying DIC and MET concentrations and ratios were prepared. Appropriate volumes of the drug stock solutions were put into 10.0 mL volumetric flasks and diluted with deionized water to the appropriate volume. The steps described in " *Construction of calibration curves* " were then carried out. The percentage recoveries were calculated using the appropriate regression equations.

### Analysis of Methoquick^®^ tablets

Methoquick^®^ tablets were precisely weighed. About 30 mL of methanol was added to a conical flask containing an amount of the properly grinded ground tablets equivalent to 50 mg of DIC and 500 mg of MET. Filtration was finished after 20 min of sonicating the contents of the flask, and the filtrate was quantitatively transferred to a volumetric flask (100.0 mL). Two additions of 30 mL of methanol were made to complete the extraction procedures. The volume was then completed to the appropriate level using the same solvent. After that, the steps described under " *Construction of calibration curves* " were carried out, and the relevant regression equations were used to determine the content of DIC and MET in their tablets.

## Results and discussion

**Fig. 2:**
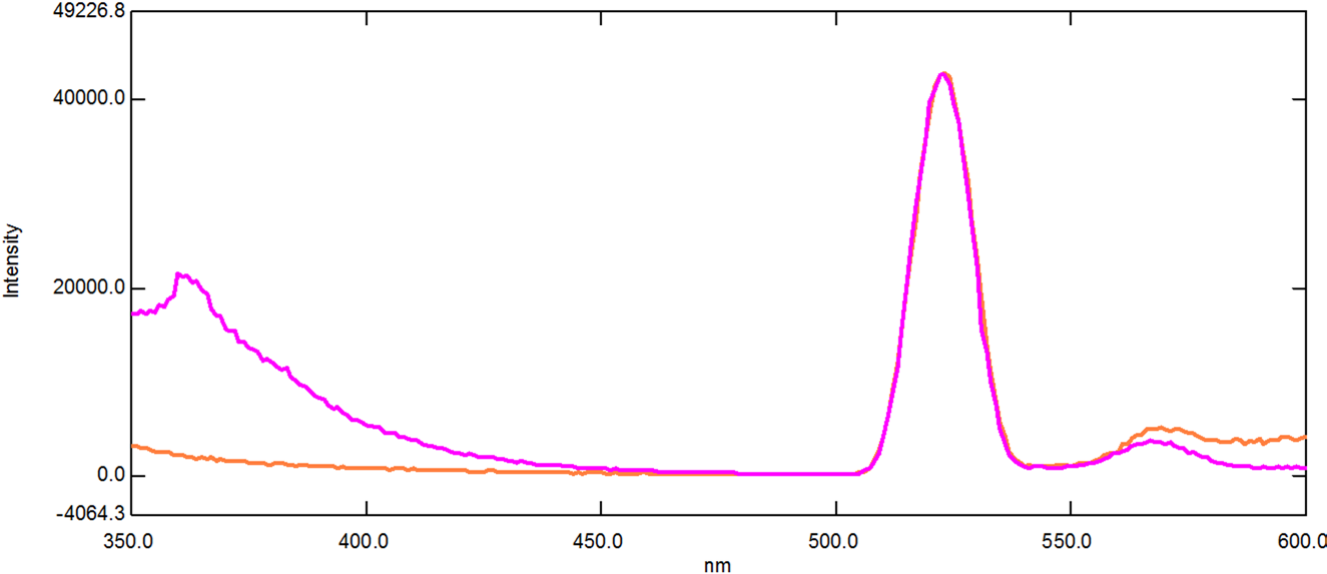
Emission spectra of 1.0 µg/mL DIC and 0.25 µg/mL MET.

**Fig. 3:**
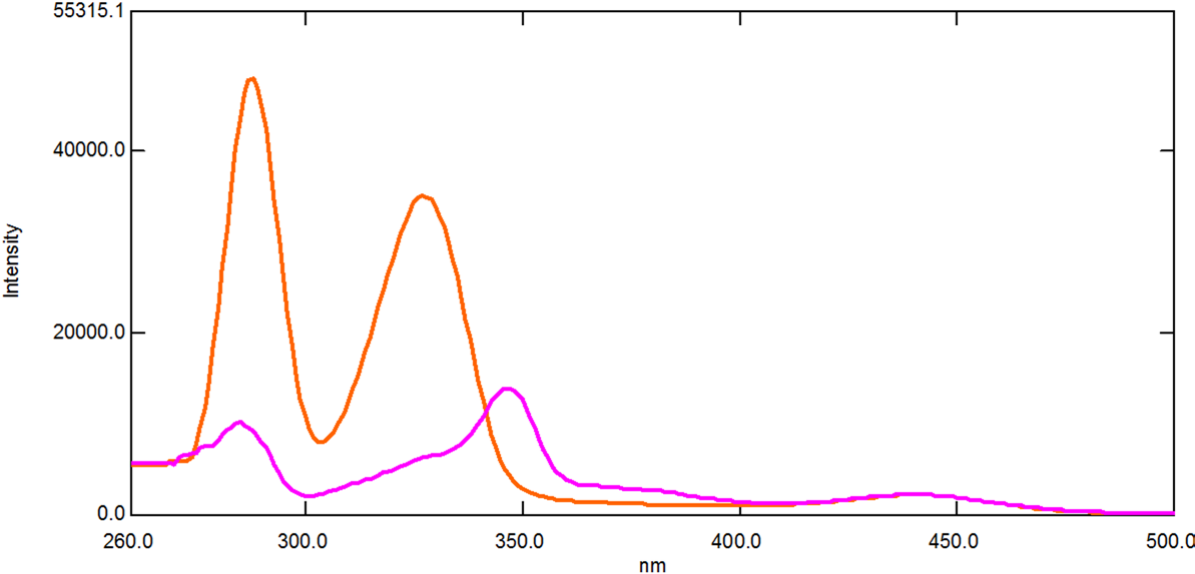
Synchronous fluorescence spectroscopy of 1.0 µg/mL DIC and 0.25 µg/mL MET at Δλ 60 nm using 1 mL phosphate buffer pH 7.

**Fig. 4:**
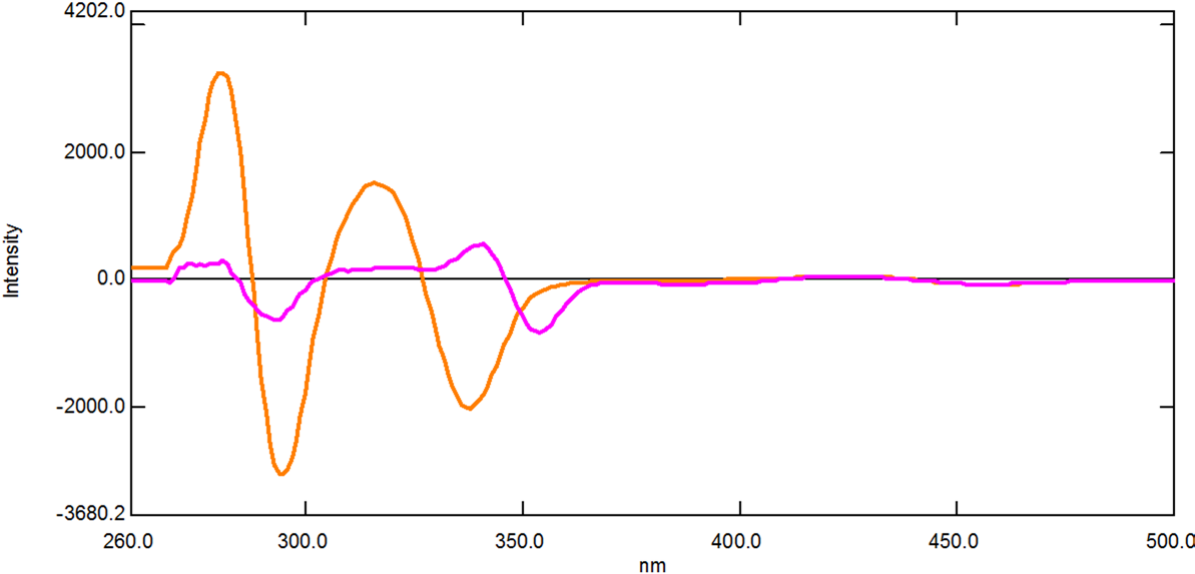
First derivative synchronous fluorescence spectra of 1.0 µg/mL DIC and 0.25 µg/mL MET at Δλ 60 nm using 1 mL phosphate buffer pH 7.

Compared to conventional fluorescence spectroscopy, SFS provides a lot of benefits, such as low interference and high selectivity^43^. SFS is a relatively straightforward and efficient way to obtain data for quantitative determination in a single run because of its resultant sharp, narrowed spectra^36^. It has been previously employed to simultaneously determine various pharmaceutical compounds binary mixtures in different matrices^44–47^. Figure 2 demonstrates that the native fluorescence spectra of DIC and MET significantly overlap, making it unfeasible to determine them simultaneously. SFS has been utilized to overcome this overlap. As shown in Fig. 3, the SFS spectra of DIC and MET still exhibit significant spectral overlap. Hence first derivative synchronous fluorescence spectroscopy (FDSFS) has been applied where DIC can be detected at 288 nm and MET at 346 nm upon using Δλ = 60 nm as Fig. 4 illustrates.

### Method optimization

The developed method’s selectivity was enhanced by carefully investigating and optimizing several experimental parameters including:

#### Selection of Δλ

**Fig. 5:**
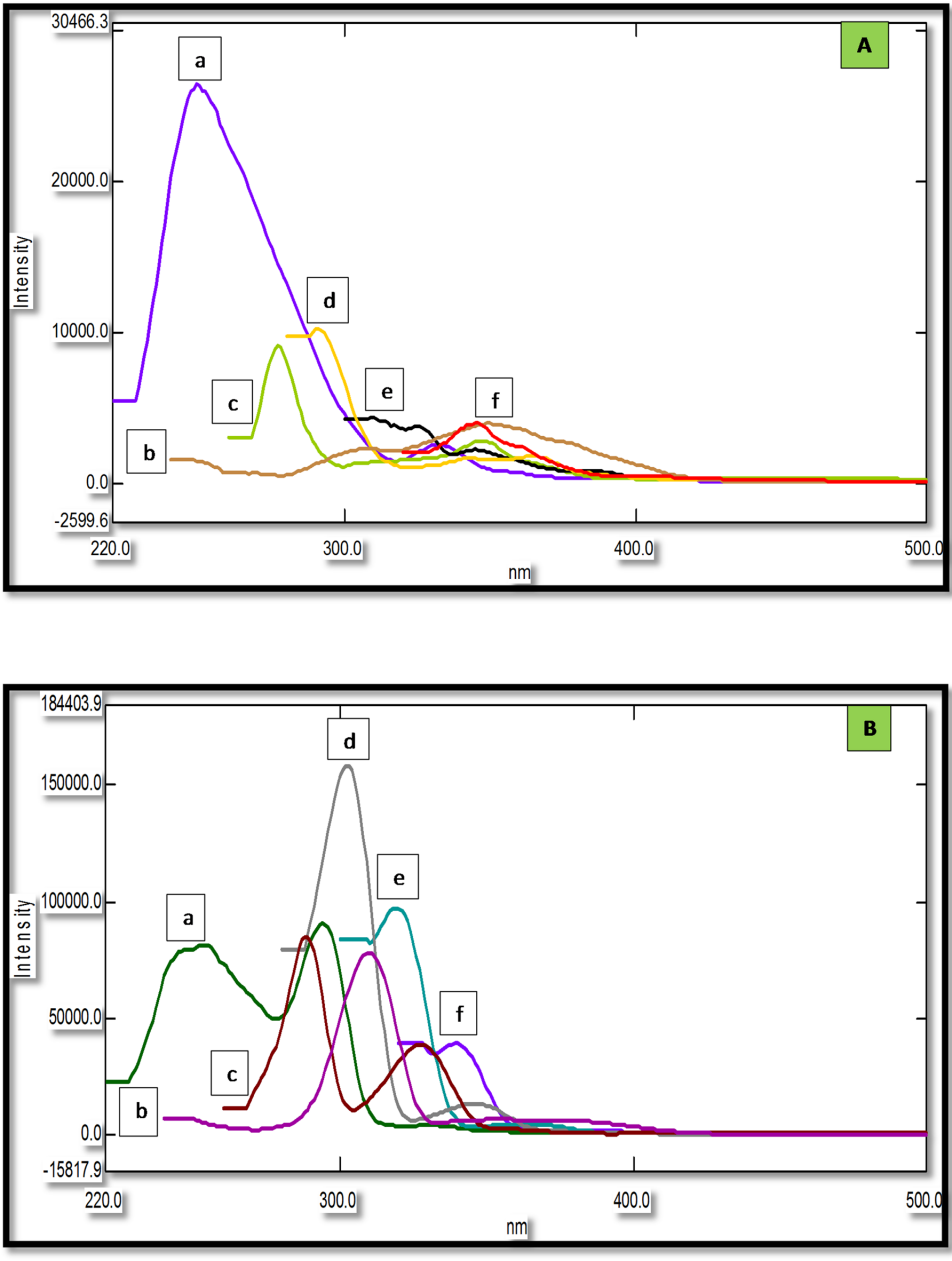
SFS of (**A**) 1.0 µg/mL DIC at Δƛ: (a) 20 nm, (b) 40 nm, (c) 60 nm, (d) 80 nm, (e) 100 nm and (f) 120 nm. (**B**) 0.25 µg/mL MET at Δƛ: (a) 20 nm, (b) 40 nm, (c) 60 nm, (d) 80 nm, (e) 100 nm and (f) 120 nm.

The choice of Δλ in CWSFS should be thoroughly studied because it has significant effect on band width and peak intensity^42^. Scanning of each of DIC and MET was performed at different values of Δλ, ranging from (20 to 120) nm. Different SFS of DIC (Fig. 5A), and MET (Fig. 5B) at various Δλ values reveal that Δλ = 60 nm is the optimum regarding peaks symmetry and resolution when simultaneously analyzed in alkaline media, providing the highest resolution and enabling DIC quantification at 288 nm following first derivatization. whereas, MET was quantified at 346 nm following first derivatization.

#### Selection of diluting solvent

A variety of solvents, including water, methanol, ethanol, acetone, and acetonitrile, were tested. The highest sensitivity and selectivity between the studied drugs have been obtained when water was used as a solvent, which adds another advantage to the suggested method.

#### Selection of buffer

Acetate buffer (pH 3-5.5) and phosphate buffer (pH 6–10) were tested. The highest sensitivity for the studied drugs has been attained when phosphate buffer (pH 7) was used.

#### Selection of buffer volume

**Fig. 6:**
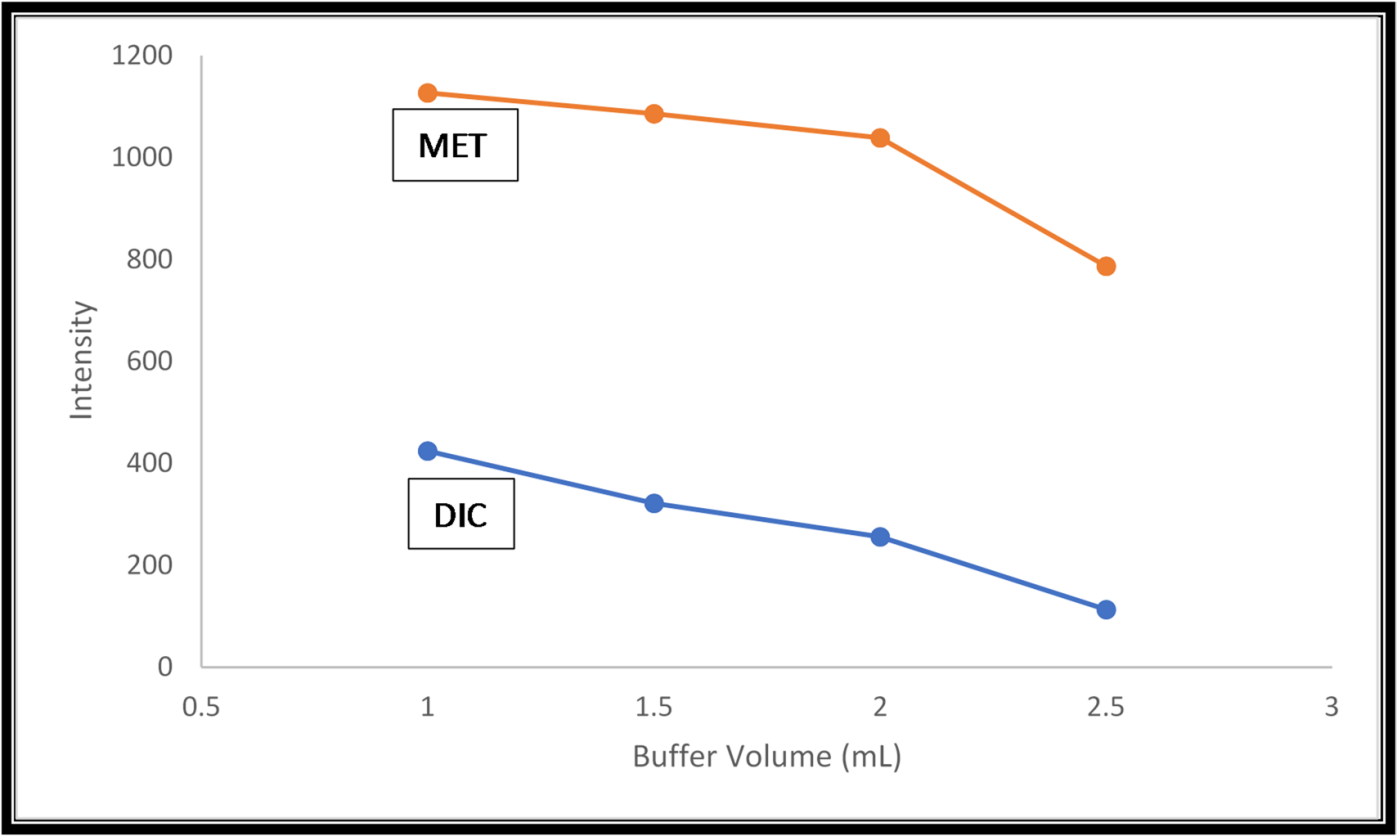
Effect of buffer volume on the intensity values (1.0 µg/mL DIC and 0.25 µg/mL MET).

Different volumes of phosphate buffer pH 7 were tried (1–3 mL), 1 mL resulted in the maximum fluorescence intensity, hence it was recommended throughout the work (Fig. 6). Different volumes below 1 mL were experimentally tried, but such volumes were not enough to reach the required pH for the prepared solutions.

### Method validation

The proposed method was fully validated according to ICH guidelines^48^ regarding linearity, range, precision, accuracy, detection limit (LOD), quantitation limit (LOQ) and robustness.

#### Linearity and range

Upon applying the proposed method DIC and MET responses were linear over the concentration ranges of 0.3–2.5 µg/mL and 0.05–5.0 µg/mL, respectively. Plotting the peak amplitudes *versus* the final concentration of each drug in µg/mL led to the construction of calibration curves, which in turn permitted the deduction of partial last square regression equations (Table 1). Low scattering of the data points around the calibration line, endorsed by correlation coefficient values that approach unity, confirms the linearity of the measured responses with concentration. Furthermore, the regression data pertaining to standard deviation of the slope (S_b_), standard deviation of the intercept (S_a_), and standard deviation of residuals (S_y/x_) were used to assess the validity of the suggested method (Table 1).

**Table 1:** Analytical performance data for the determination of diclofenac and methocarbamol applying the proposed method.

Parameter	Diclofenac	Methocarbamol
Concentration range (µg/mL)	0.3–2.5	0.05–5.0
Limit of detection LOD (µg/mL)	0.156	0.083
Limit of Quantitation LOQ (µg/mL)	0.471	0.251
Regression equation	Y = 160.77x + 375.63	Y = 3571x + 171.93
Correlation coefficient (r)	0.9971	0.9994
S.D. of the residuals (S_y/x_)	9.52	167.8
S.D. of the intercept (S_a_)	7.58	89.8
S.D. of the blank (ϭ)	1.421	1.276

#### LOQ and LOD

The lowest drug concentration that can be identified under the stated experimental conditions but not necessarily quantified is known as (LOD)^48^. The lowest analyte concentration that can be identified with acceptable accuracy and precision is known as the (LOQ)^48^. The calculated values are listed in (Table 1).

#### Precision

The intra-day and inter-day precision were evaluated using three replicate analyses of three concentrations of pure DIC and MET within their linearity ranges on the same day or on three consecutive days respectively. The obtained results are summarized in (Table 2). The small SD values demonstrate the high precision of the proposed approach.

**Table 2:** Precision data for the determination of diclofenac and methocarbamol applying the proposed method.

Drug	Intra-day precision		Inter-day precision	
	Conc. Taken (µg/mL)	Mean* %found ± SD	Conc. Taken (µg/mL)	Mean* %found ± SD
Diclofenac	0.3	99.23 ± 0.12	0.3	98.95 ± 0.28
	1.0	98.71 ± 0.22	1.0	97.72 ± 0.42
	2.5	99.41 ± 0.17	2.5	100.73 ± 0.25
Methocarbamol	0.25	99.72 ± 0.11	0.25	99.23 ± 0.31
	1.0	100.39 ± 0.15	1.0	100.62 ± 0.11
	3.0	101.23 ± 0.31	3.0	101.28 ± 0.28

*Each result is the average of three separate determinations.

#### Accuracy

Results obtained from drug analyses applying the proposed method in pure form and in laboratory prepared mixtures (Table 3) showed acceptable accuracy as revealed from the percent recoveries obtained. By comparing the results obtained upon application of the proposed method to the analysis of the analytes in their tablets with those of comparison method^49^, it is possible to conclude that the proposed method is accurate as illustrated from the low *t* and *F* test values^50^.

**Table 3:** Assay results for the determination of diclofenac and methocarbamol in pure form and in laboratory prepared mixture applying the proposed method.

Parameters		Diclofenac			Methocarbamol		
		Conc. Taken (µg/mL)	found	% found	Conc. Taken (µg/mL)	found	%found
Pure form		0.3	0.298	99.3	0.25	0.246	98.4
		0.5	0.508	101.6	1	1.007	100.7
		1	0.985	98.5	2	2.001	100.05
		2	2.03	101.5	3.5	3.496	99.9
		2.5	2.481	99.2	5	5.001	100.02
Mean ± SD				100.03 ± 1.42			99.81 ± 0.85
Ratio DIC: MET	Laboratory Prepared mixture						
	1: 10	0.3	0.292	97.3	3.0	2.993	99.8
	1: 2	0.7	0.699	99.9	1.4	1.417	101.2
	1: 1	1.0	0.983	98.3	1.0	0.994	99.4
	2: 1	1.2	1.195	99.6	0.6	0.592	98.7
	0.5: 1	1.3	1.307	100.5	2.6	2.601	100.04
Mean ± SD				99.12 ± 1.29			99.81 ± 0.93

#### Robustness

Consistency of peak amplitudes with deliberately minor changes in various experimental conditions was used to establish it. These changes related to method and involve the buffer volume (1 mL ± 0.1) as shown in (Fig. 6). The robustness of the method was demonstrated by the fact that peak amplitudes of the drugs under study were not influenced by these little changes that may happen during the experimental process.

### Applications

#### Application of the proposed method to dosage form

Diclofenac and methocarbamol in its tablets were effectively analyzed using the suggested method, the results exhibited were in good agreement with those obtained *via* the reference HPLC method which used a C18 column with mobile phase composed of phosphate buffer: methanol (30:70), adjusted to pH 4.5 with orthophosphoric acid, adopting a flow rate 1 mL/min., with UV detection at 281 nm^49^. The calculated *t* and *F* values were less than the tabulated ones, which highlighted the correspondence between the suggested and reference method^50^. Table 4 provides an overview of the obtained data.

**Table 4:** Application of the proposed method to the analysis of diclofenac and methocarbamol in Methoquick^®^ tablets.

Parameters	Diclofenac			Methocarbamol		
	Conc. Taken (µg/mL)	%found	Comparison method	Conc. Taken (µg/mL)	%found	Comparison method
Methoquick^®^ tablets	0.3	99.69	100.5	3.0	99.71	100.55
	0.35	100.27	99.82	3.5	98.63	100.07
	0.4	99.76	99.61	4.0	99.99	99.75
	0.45	100.62	99.82	4.5	99.78	99.18
	0.5	99.63	100.31	5.0	101.42	100.54
Mean ± SD		99.99 ± 0.43	100.012±0.38		99.9 ± 0.99	100.018±0.58
*t* test		0.957 (2.776)*			0.812 (2.776)*	
*F* test		1.280 (6.388)*			2.913 (6.388)*	

*Tabulated *t* and *F* values at *p* = 0.05^50^.

## Greenness assessment of the proposed method

**Fig. 7:**
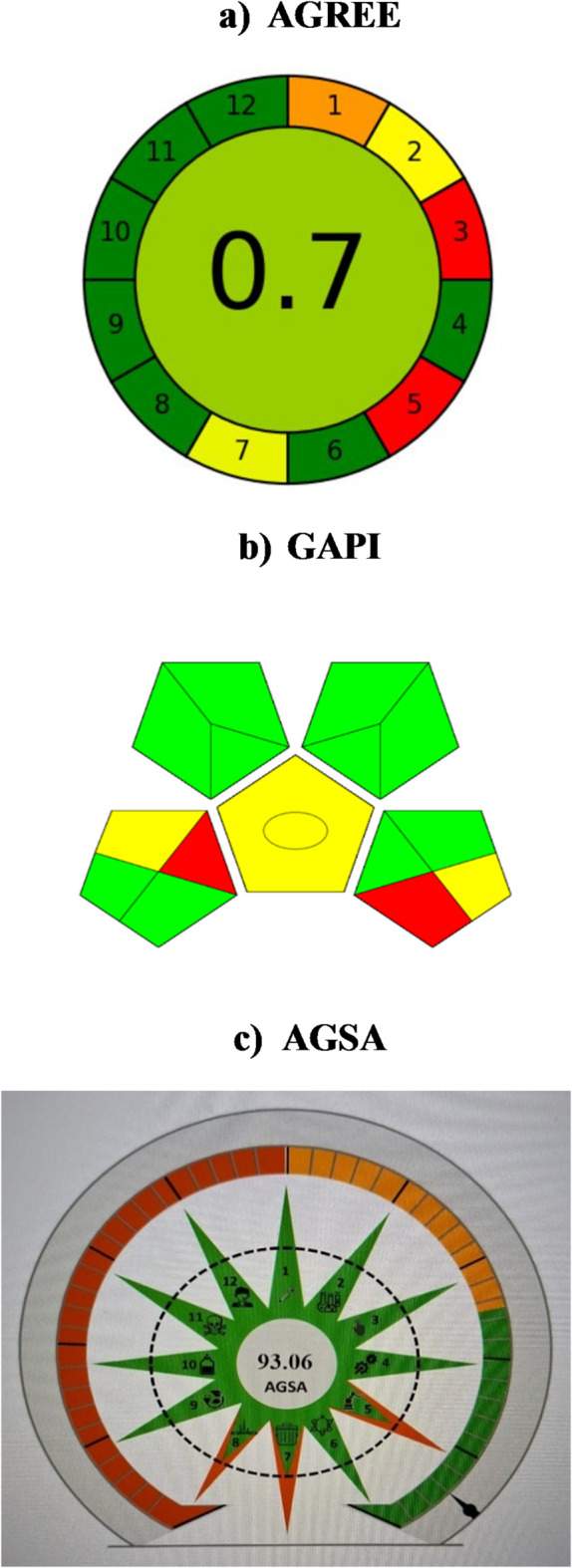
Assessment of the greenness profile of the proposed method by AGREE (**a**), GAPI (**b**) and AGSA **(c)** metrics.

AGREE^51^, GAPI^52^, and AGSA^53^ were used to assess the proposed method’s greenness. AGREE is a metric system used in the proposed method to determine environmental and occupational hazards. It assesses twelve important environmental criteria and assigns a score between 0 and 1 to represent how environmentally friendly the analytical process is^51^. On the other hand, GAPI symbolically express the greenness of individual aspects of the analytical procedure *via* three levels of color scale: green, yellow, or red, indicating high, medium, or low impact respectively^52^. Eventually, the Analytical Green Star Area (AGSA) presents a thorough, integrated rating system that is easy to use. AGSA ensures objective evaluation while adhering to the 12 GAC Principles by integrating method classification, integrated scoring, and resistance to human prejudice^53^. Our proposed method is superior in terms of greenness compared to the reported chromatographic method^49^, as we use spectrofluorimetry, which uses less energy, produces less waste, uses a safer solvent, and takes less time than HPLC. Consequently, the method was observed to be excellent green with a green GAPI pictogram along with higher AGREE and AGSA scores as demonstrated in Fig. 7.

## Conclusion

In order to determine DIC and MET in their combined dosage form simultaneously, this work showed how to develop and validate a sensitive, accurate, specific, and precise FDSFS approach accomplishing LOD values 0.15 and 0.03 µg/mL, respectively. Furthermore, the developed method has an economical advantage and do not require complex requirements for sample and data processing. In addition to the reproducibility as well as the simplicity and convenience. So it can be applied in quality control laboratories for analysis of the studied drugs. Applying the procedure to Methoquick^®^ tablets is encouraged by its high sensitivity.

## Data Availability

The datasets generated and/or analyzed during the current study are available from the corresponding author upon reasonable request.
